# ^1^H NMR- based metabolomics approaches as non- invasive tools for diagnosis of endometriosis

**Published:** 2016-01

**Authors:** Negar Ghazi, Mohammad Arjmand, Ziba Akbari, Ali Owsat Mellati, Hamid Saheb-Kashaf, Zahra Zamani

**Affiliations:** 1 *Department of Biochemistry, Zanjan Medical University, Zanjan, Iran.*; 2 *Department of Biochemistry, Pasteur Institute of Iran, Tehran, Iran.*; 3 *Navid Medical Center, Tehran, Iran.*

**Keywords:** *Metabolomics*, *Endometriosis*, *Nuclear magnetic resonance*

## Abstract

**Background:**

So far, non-invasive diagnostic approaches such as ultrasound, magnetic resonance imaging, or blood tests do not have sufficient diagnostic power for endometriosis disease. Lack of a non-invasive diagnostic test contributes to the long delay between onset of symptoms and diagnosis of endometriosis.

**Objective:**

The present study focuses on the identification of predictive biomarkers in serum by pattern recognition techniques and uses partial least square discriminant analysis, multi-layer feed forward artificial neural networks (ANNs) and quadratic discriminant analysis (QDA) modeling tools for the early diagnosis of endometriosis in a minimally invasive manner by ^1^H- NMR based metabolomics.

**Materials and Methods:**

This prospective cohort study was done in Pasteur Institute, Iran in June 2013. Serum samples of 31 infertile women with endometriosis (stage II and III) who confirmed by diagnostic laparoscopy and 15 normal women were collected and analyzed by nuclear magnetic resonance spectroscopy. The model was built by using partial least square discriminant analysis, QDA, and ANNs to determine classifier metabolites for early prediction risk of disease.

**Results:**

The levels of 2- methoxyestron, 2-methoxy estradiol, dehydroepiandrostion androstendione, aldosterone, and deoxy corticosterone were enhanced significantly in infertile group. While cholesterol and primary bile acids levels were decreased. QDA model showed significant difference between two study groups. Positive and negative predict value levels obtained about 71% and 78%, respectively. ANNs provided also criteria for detection of endometriosis.

**Conclusion:**

The QDA and ANNs modeling can be used as computational tools in noninvasive diagnose of endometriosis. However, the model designed by QDA methods is more efficient compared to ANNs in diagnosis of endometriosis patients.

## Introduction

Endometriosis, is defined by the presence and growth of resembling endometrial tissue outside the uterus (1). Endometriosis affects an estimated 176 million women worldwide usually between the age of 15-49 years, in whom remain undiagnosed and are therefore not treated (2). At present, the gold standard method to diagnose endometriosis is visual inspection by laparoscopy and biopsy of the tissue. However, this is an expensive and invasive procedure. 

Diagnosing endometriosis is also a challenge, since only expert gynecologist may diagnose the disease. The extensive efforts made to improve its diagnosis with noninvasive methods that can be justified by in accuracy of clinical examination, and the risks and the diagnostic limitations of laparoscopy. There has been a strong interest in the pathogenesis of endometriosis that is why; new technologies- such as omics science- have emerged to focus on this disease through the use of genomics, proteomics or metabolomics. Metabolomics is a relatively new field of study that focuses on small molecule metabolites (3). 

Metabolomics profiling has emerged as a powerful and reliable tool for the identification of total metabolites present in different biological systems under a given physiological condition and changes in metabolite content/ concentration are amplified relative to changes in the transcriptome and proteome (4, 5). The artificial neural network (ANNs), with supervised pattern recognition method, quadratic discriminant analysis (QDA) are sophisticated computational modeling tool that proposed to diagnose the disease with prediction signs in combination with metabolomics data set (6, 7).

In this study, it was hypothesized that metabolic profiles of endometriosis patients are changed compared with healthy group. Proton Nuclear Magnetic Resonance (^1^H-NMR) Spectroscopy is one of the popular spectroscopic techniques, which can be used to obtain rich signal to identify and quantify complex mixtures of metabolites with little or no sample preparation. Furthermore, only small sample volumes are required, and the test is nondestructive (8).

## Materials and methods

**Study design and sample source**

This prospective cohort study was done in Pasteur Institute, Iran in June 2013. Ethic Committee of the Research Center of Metabolic Disease of Zanjan University of Medical Sciences approved the study protocol. Written informed consent was taken from all participants. 31 randomized infertile women with endometriosis (stage II and III) aged 22-44 years who confirmed by diagnostic laparoscopy in Navid clinical center, Tehran, Iran were enrolled as case group. The control group was consisted of 15 randomized normal women in the same age range. They (control group) had no pelvic pain, pelvic inflammatory disease and base on diagnostic laparoscopy there were no signs of endometriosis.

These women were referred to the infertility center due to male factor infertility. Our exclusion criteria were followed as: receiving any kind of medical or hormonal treatment during the last two months, history of gynecological disorders or surgery. Staging was done according to the American Society for Reproductive Medicine classification of endometriosis (9). Most patients had endometriosis symptoms for six months priors to laparoscopic surgical procedure. 

Fasting (more than 8 hr) venous blood samples (5 ml) were collected from all participants in early follicular phase during daytime. Considering the effects of estrogen, it was postulated that the scale of the responses would be greater when measured during the early follicular phase compared to the rest of the menstrual cycle. Blood was allowed to clot and serum was separated by centrifugation at 3,000 rpm for 5 min at 4^o^C were stored at -80^o^C until ^1^H NMR analysis.

^1^ **H Nuclear magnetic resonance spectroscopy**

Prior to the ^1^H-NMR experiment, frozen serum samples were thawed at room temperature and vortexted. 540 μL serum was mixed with 60 μL of deuterium oxide (D2O- Pad, kimia Novin Company, Iran) with 3-trimethylsilyl- 1- propanesulfonic acid sodium salt (TMSP- Sigma Company, USA) as internal standard for each sample. ^1^H-NMR spectra were obtained using Carr- Purcell- Meiboom-Gill (CPMG) protocol in Bruker 400 MHz instrument. 

Water peak was suppressed and 128 scan were taken with relaxation time of 3 seconds at 295.5 K temperature for each sample as described earlier (10). Preprocessing of spectra included spectral alignment, baseline correction, elimination of uninformative spectral regions and normalization was done with ProMetab toolbox in MATLAB software (11). The water signal region at 4.7 ppm and regions between 5.4-6.7 ppm, which contained no significant metabolite signal were eliminated (12).

**Metabolites and pathway analysis**

Expression of different metabolites in endometriosis compared to the controls was analyzed using partial least squares discriminant analysis using MetaboAnalyst 3.0, a web-based analytical pipeline for high-throughput metabolomics studies. The analysis was applied to 46 spectra from 31 endometriosis and 15 control serum samples. The regression coefficient and variable importance in projection score were obtained and then used to identify the metabolites (chemical shifts) contributed most to this classification. 

**Figure 1:**
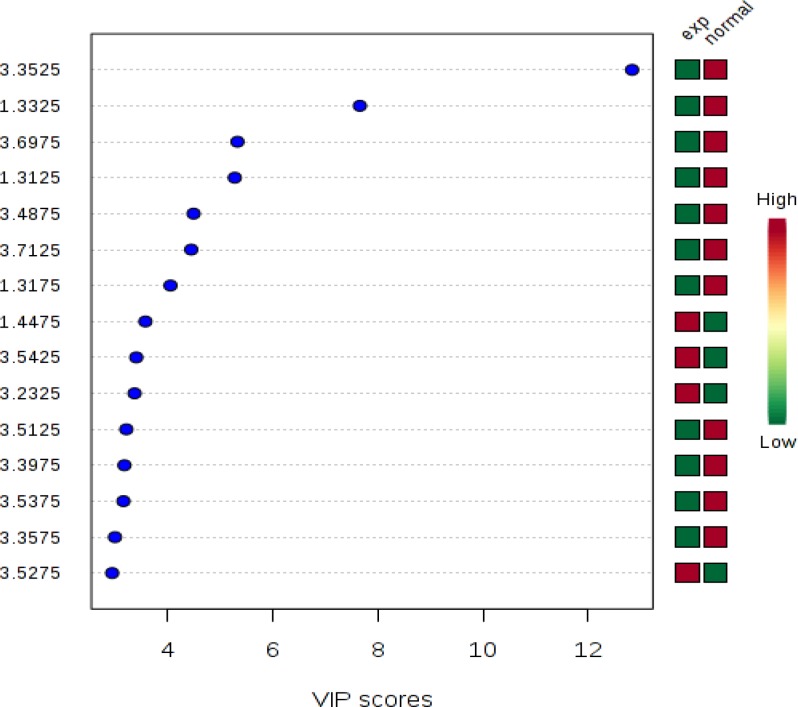
Fifteen most important metabolites binned identified by PLS- DA. The colored boxes on the right indicate the relative concentrations of the corresponding metabolite in each group under study. Green color shows decreases and red color shows the increase in concentration of metabolites

To determine separated metabolites, Human Metabolome Database (HMDB) was used. Chemical shift was feed and corresponding metabolite found out (Figure 1). 

Metabolic pathway analysis (Metaboanalyst version 3) was used to assess biochemical pathways that were most affected by endometriosis disease. 

**Classification model**

QDA as a quadratic boundary based classifier and feed forward artificial neural networking (ANNs), which is multilayer classifier were utilized as tools to diagnose the patients with endometriosis and controls. The ANNs model was built using multilayer perceptron with all spectra’s chemical shift between 0-5.5 ppm as inputting data. The model was taught in maximum of 1000 iteration, with twenty neurons in hidden layer and two outputs by Tanagra statistical package. 

Performance of the built model was evaluated using 10- fold cross- validation and non- error rate (percent of correctly classified samples) of the validation was reported. For QDA classifier, correct classification rate, positive predictive value, and negative predictive value were estimated to reveal the performance of the classification.

**Statistical analysis**

Data were analyzed using Student’s t-test by Statistical Package for the Social Sciences, version 16.0, SPSS Inc, Chicago, Illinois, USA (SPSS software).

## Results

^1^ **H- NMR spectroscopy and pattern recognition analysis**

**Figure 2:**
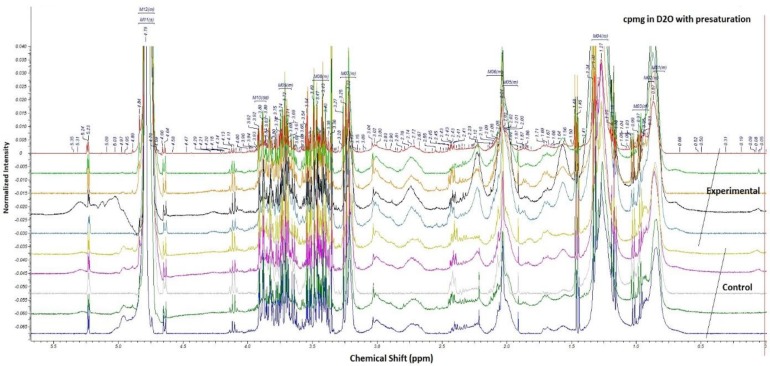
Representative 400 MHz ^1^H- NMR spectra of serum samples from five controls and five cases. X axis shows the chemical shift of metabolites between 0-5.5 ppm. Y axis shows the intensity of each metabolite peak

Spectra of serum samples showed the variation in spectra between two groups (Figure 2). Most altered metabolic pathways are shown in table I and II. ANN analysis for case group consist training and validation steps. 70% of group observation were selected for training and the remained 30% were verified in the model. The results showed that all 11 samples in training group were recognized as normal and 21 samples in patient group classified as patient, all samples were correctly classified and error rate was zero (Table III). We examined the sensitivity of our model with 30% of remaining samples. Sensitivity discriminate between groups was 50% with error rate of 0.4286 (Table IV). 

**Figure 3:**
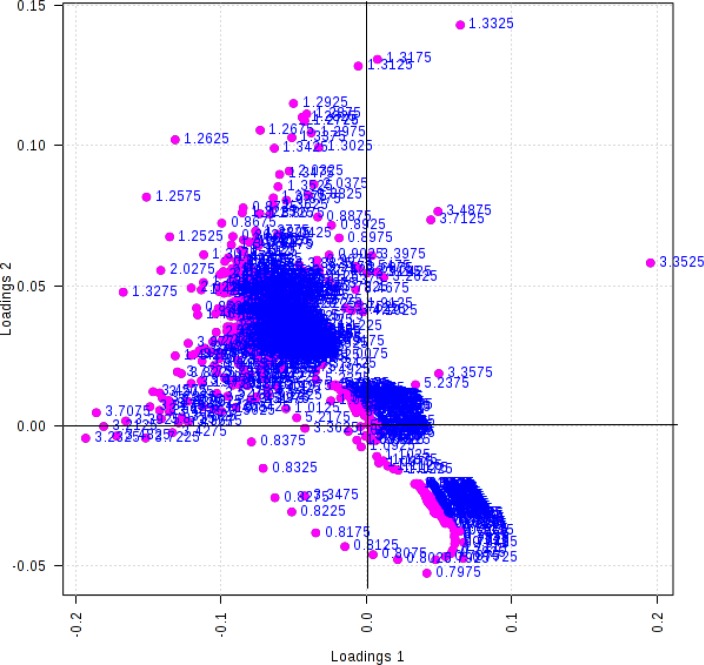
PLS- DA loading plot (Dots represent the NMR chemical shift of metabolites binnes, Dotes accumulates at center show the similarity of metabolites ppm in two groups, where dotes spotted on distances from centers shows the significant outliers in two group of study

A 10-fold cross validation was performed (10% of samples left out randomly) to evaluate the performance of QDA classifier. Mean values of classification results for five times replication of 10-fold cross validation are reported in Table V. PLS-DA result shows the good classification between groups with loading plot (Figure 3). 

Metabolic pathways relating to these separated metabolites were studied by the help of metabolomics data analyzing (www.metaboanalyst.ca) database.

**Table I:** Changed metabolites in two groups

**Serial Number**	**Meatbolites name**	**Human metabolome database number**
1	2-Methoxyestrone	HMDB00010
2	Deoxycorticosterone	HMDB00016
3	7-Dehydrocholesterol	HMDB0032
4	Taurocholic acid	HMDB00036
5	Aldosterone	HMDB00037
6	Androstenedione	HMDB00010
7	Cholesterol	HMDB00016
8	Dehydroepiandrosterone	HMDB00032
9	2-methoxyestradiol	HMDB00036

**Table II:** Pathways that were altered in patients with endometriosis compared to controls

**Biochemical pathways**	**Total**	**Hits**	**Raw P**	**FDR**
Steroid hormone biosynthesis	99	6	31×10^-8^	25×10^-6^
Primary bile acid biosynthesis	47	2	123×10^-4^	493×10^-3^
Biotin metabolism	11	1	405×10^-4^	1
Taurine and hypotaurine metabolism	20	1	725×10^-4^	1

Total: the total number of compounds in the pathway; Hits: the actually matched number from the user uploaded data; Raw p: the original p value calculated from the enrichment analysis; FDR p: the p value adjusted using False Discovery Rate.

**Table III:** Multilayer feed forward neural networks classification modeling

**Classifier performances**						
**Values prediction**			**Confusion matrix**			
Value	Recall	1-Precision		Normal	Exp	Sum
Normal	1.0000	0.0000	Normal	11	0	11
Exp	1.0000	0.0000	Exp	0	21	21
			Sum	11	21	32

Confusion matrix contains training of seventy percent of experimental and control group. Error rate is frequency of errors, Recall is sensitivity and 1-Precision is specificity rate, which shows 100 percent sensitivity and specificity.

Error rate= 0.000

**Table IV:** Multilayer feed forward neural networks Classification prediction test using 30% sample

**Prediction supervised instance**						
**Values prediction**			**Confusion matrix**			
**Value**	**Recall**	**1-Precision**		**Normal**	**Exp**	**Sum**
Normal	0.7500	0.6250	Normal	3	1	4
Exp	0.5000	0.1667	Exp	5	5	10
			Sum	8	6	14

Error rate is frequency of errors, Recall is sensitivity and 1- Precision is specificity rate. Sensitivity of our control group was 75 percent with 63 percent specificity and 50 percent sensitivity for experimental group with 17 percent specificity.

Error rate= 0.4286

**Table V:** Quadratic Discriminant Analysis Classification modeling

**Trail**	**Correct classification rate**	**Positive predictive value**	**Negative predictive value**
First	0.75	0.67	0.79
Second	0.78	0.75	0.8
Third	0.76	0.71	0.79
fourth	0.75	0.69	0.77
fifth	0.76	0.73	0.77
mean	0.76	0.71	0.78

Mean of quadratic discrimination modeling show 76% correctness. Positive predictive value is the probability of truly diagnosed patient with endometriosis, i.e. among those who had a positive screening test, the probability of disease was 71%. Negative predictive value is the probability of truly diagnosed person with no diseases. The sensitivity of this group test is 78%.

**Table VI:** Comparison between quadratic discriminant analysis QDA and artificial neural networking ANNs classification

**Analysis**	**Correct classification**	**Positive predict value**	**Negative predict value**
QDA	76%	71%	78%
ANN	58%	50%	75%

Sensitivity discrimination of two group by QDA is 76%, whereas by ANN is 58%. As seen in table positive and negative predict value is also more sensitive by QDA rather than ANN.

QDA: quadratic discriminant analysis

ANN: artificial neural networking

## Discussion

In the present investigation, we applied a ^1^HNMR- based metabolome profiling approach to investigate metabolic changes in serum from endometriosis patients and model the discriminated metabolites for early diagnose of endometriosis. In this regard, several metabolites including 2-mthoxyestradiol, 2- methoxyestrone, dehydroepiandrostion, androstendione, and cholesterol showed significant increase in the endometriosis group in comparison with the control group. 

Meanwhile in juxtaposition, primary bile acids metabolite decreased significantly in the endometriosis group. Generally, there are two ways to synthesize estradiol. The first is to synthesize it from cholesterol and the second is through aromatase enzymes, which convert androgens into the estrogens estradiol (skin and adipose tissue). Various studies have found that in endometriosis, the increase of aromatase enzyme gives rise to increase of the levels of estradiol in adipose tissue and peripheral tissues (13-15). This increase in serum estradiol levels of these patients is not measurable and is usually in the normal range (16). 

The increase of the estradiol metabolites, namely 2- methoxyestrone and 2-methoxyestradiol, in serum and urine can be indicative of increase of estradiol in the body. The increase of these metabolites, while the level of estradiol is normal (unpublished data), can be attributed to the increase of estradiol in the peripheral tissues due to aromatase enzyme. Slow and dormant progress of endometriosis is attributed to antiangiogenic properties of 2-methoxyestrone and 2- methoxyestradiol. 

This property might be useful in control and treatment of endometriosis (17). In the current study, the levels of these two metabolites were increased. Miller *et al* found that dehydroepiandrostion (DHEA) metabolites could activate estrogen's alpha and beta receptors thus it leads to increasing estradiol and its metabolites. They found that DHEA and its metabolites are possible contributors in increasing risk of breast cancer in postmenopausal women (18). In the current study, DHEA and androstendione were increased in endometriosis patients. 

This increase can be attributed to the stimulant effects of these two metabolites of estradiol. Jana *et al* recent study have found that oxidative stress is increased in the patients with advanced endometriosis (19). Turgut *et al* and Melo *et al* compared advanced stage endometriosis patients with the control group without endometriosis with respect to the oxidative stress markers and found an increase in oxidative stress, which resulted in an increase in total cholesterol in women with advanced stage endometriosis (20, 21). The majority of estrogen is excreted through the liver by primary bile acids and its smaller amount is excreted though the urine (22). 

In this study, it was found that reduction in primary bile acids might cause hyperestrogenism as a result of failure of hepatic removal of estrogens from the circulation and consequently increasing growth ectopic endometriosis. In this investigation, the endeavor was to find a new NMR-based metabolome profile marker (outliers) in endometriosis patients through serum metabolites, which are directly or indirectly related to endometriosis disease and model and calibrate the model for early prognosis of the disease. There are many reports concerning the use of ANNs in gynecological diagnosis. Sristatidis *et al* postulated that ANNs could be used as promising tools in early diagnosis of these types of diseases. 

ANNs’s prediction power was also evaluated in in vitro fertilization by the same team (23). Lapeer and colleagues used ANNs for predictive tasks, to find out perinatal parameters influencing birth weight (24). In similar investigation, Atkov *et al* reported >90% accuracy in diagnosing coronary heart diseases (25). To our knowledge, this is the first report that ANNs and QDA could be used in endometriosis assessments with metabolome profile data.

## Conclusion

In conclusion, the models built by QDA and ANNs showed that the two modeling tools can identify serum levels in the patients with endometriosis with 71% and 50% precision, respectively. However, the model designed by QDA methods is more efficient in recognizing endometriosis patients compared to ANNs.

## Note

***This article extracted from M.Sc. thesis. (Negar Ghazi)***
